# Supplementary material for: Reconciling global mean and regional sea level change in projections and observations
Source: Nat Commun. 2021 Feb 12;12:990. doi: 10.1038/s41467-021-21265-6 (PMC7881246; doi:10.1038/s41467-021-21265-6)
Supplement: Supplementary file 1 — Supplementary Information [file 41467_2021_21265_MOESM1_ESM.pdf]

Supplementary Information

Reconciling global mean and regional sea level change in projections and observations

Jinping Wang[1,2], John A. Church[3*], Xuebin Zhang[2*] and Xianyao Chen[4]

[1] Department of Oceanography, College of Oceanic and Atmospheric Sciences, Ocean University of China, China

[2] Centre for Southern Hemisphere Oceans Research (CSHOR), CSIRO Oceans and Atmosphere, Hobart, Australia

[3] Climate Change Research Centre, University of New South Wales, Sydney NSW 2052, Australia

[4] Frontiers Science Center for Deep Ocean Multispheres and Earth System, Key Laboratory of Physical Oceanography, Ocean University of China, Qingdao, China

* Corresponding authors. E-mail: john.church@unsw.edu.au; xuebin.zhang@csiro.au

Supplementary Figures

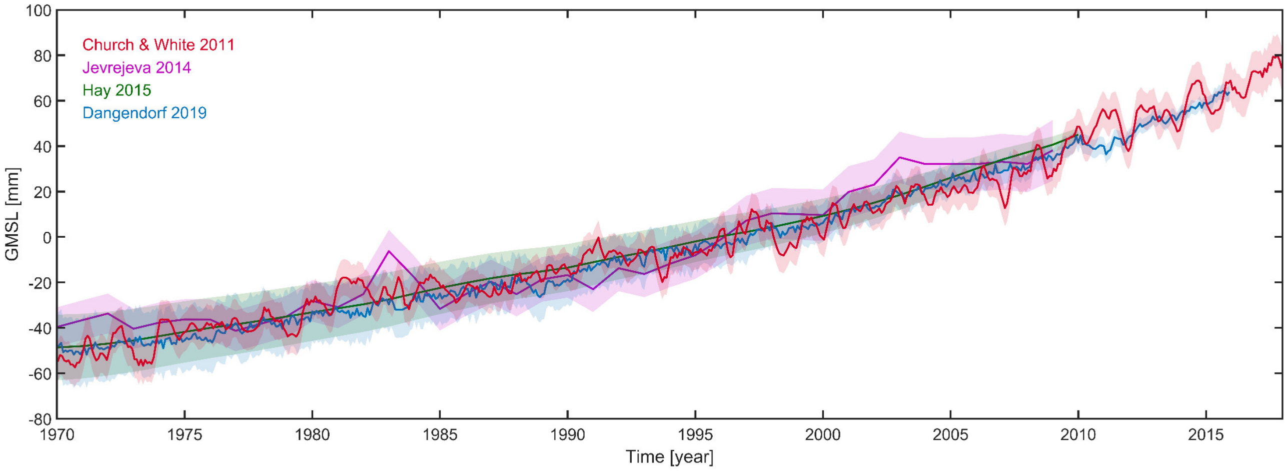

Supplementary Figure 1. The global mean sea level (GMSL) reconstructions since 1970.

The GMSL reconstruction are from ref. [1] (red), ref. [2] (purple), ref. [3] (green) and ref. [4] (blue) including the glacial isostatic adjustment (GIA) signal. GMSL from refs. [2,3] are available at annual resolution. The shading denotes the estimated error on the 1 standard deviation level.

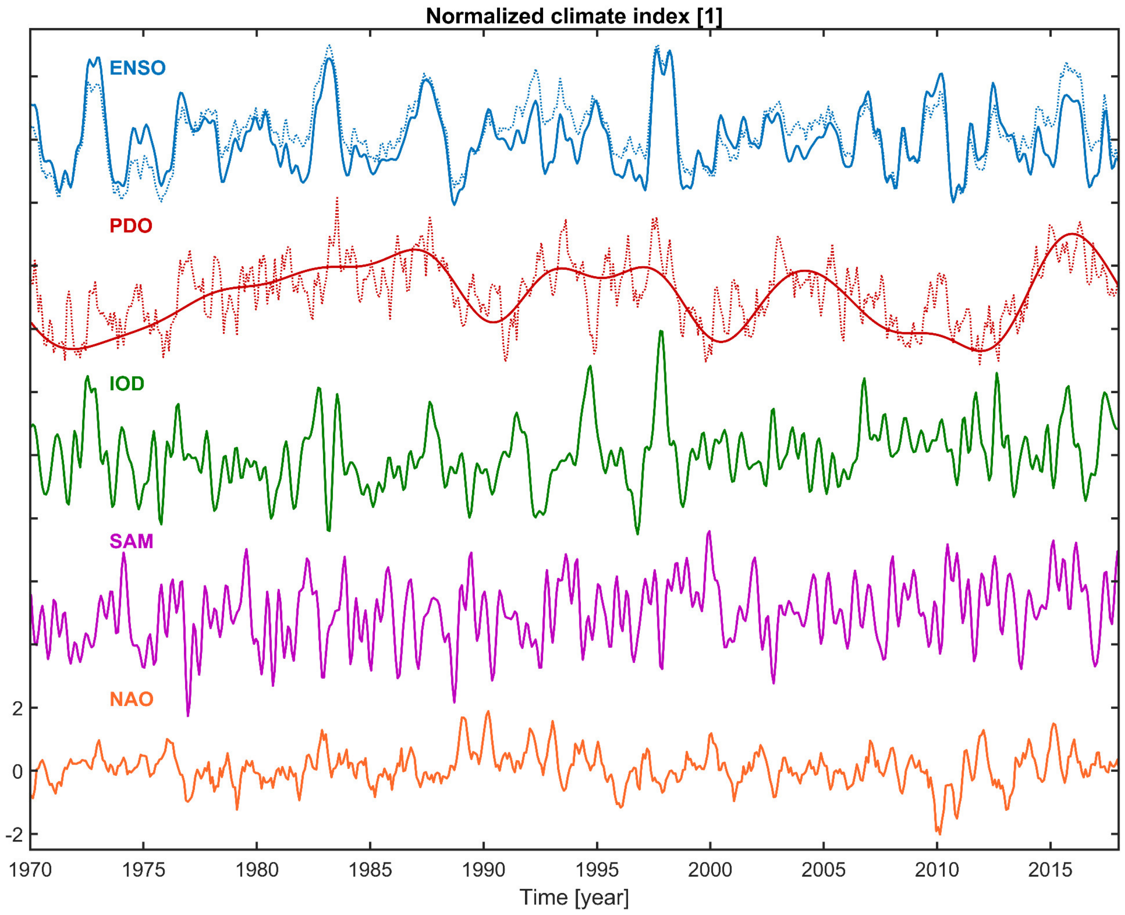

Supplementary Figure 2. The normalized climate indices used in the MVLR analysis. The high-pass filtered El Niño - Southern Oscillation (ENSO; blue) and low-pass filtered Pacific Decadal Oscillation (PDO; red) indices, the dashed lines are corresponding unfiltered time series. The smoothed the Indian Ocean Dipole (IOD; green), the Southern Annular Mode (SAM; purple) and North Atlantic Oscillation (NAO; orange) indices with a 5-month running filter. The units are 1.

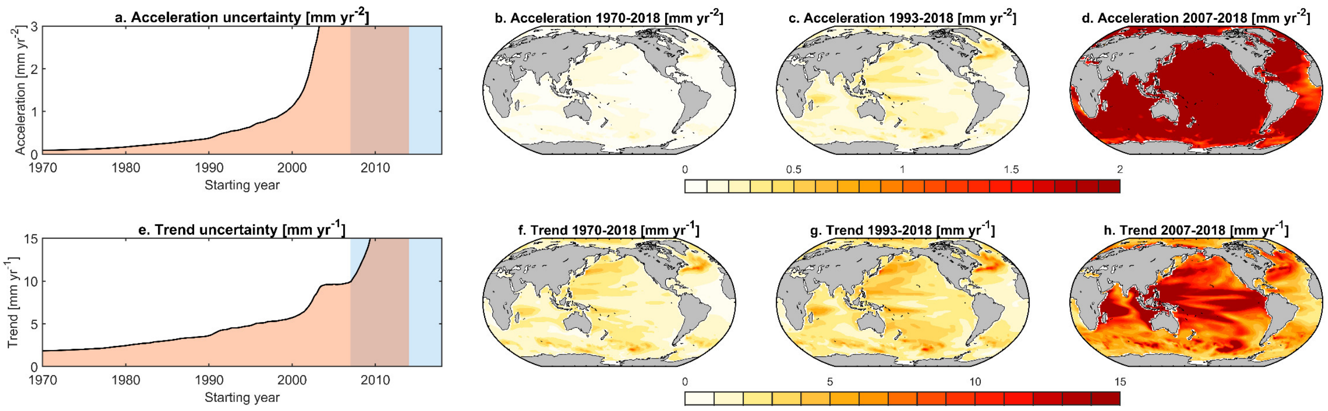

Supplementary Figure 3. Trend and acceleration uncertainties (90% confidence level) over historical period. The dynamic sea level (DSL) are from the Community Earth System Model (CESM) large ensemble developed by the National Center for Atmospheric Research (NCAR). The natural variability is removed via multiple variable linear regression (MVLR) with fixed ending year of 2018 but changed starting years, ranging from 1970-2013. **a**, The orange shading indicates the historical acceleration uncertainty [mm yr^{-2}] which is estimated by the standard deviation (STD) of 35 realizations. Values along the x-axis indicate the starting year. The blue shaded area indicates the overlapping period between observations and projections (2007-2018). **b-d**, The spatial map of acceleration uncertainties [mm yr^{-2}] with natural variability removed during 1970-2018, 1993-2018, and 2007-2018 respectively. **e-h**, Same as (**a-d**) but for linear trend [mm yr^{-1}] during the overlapping period 2007-2032.

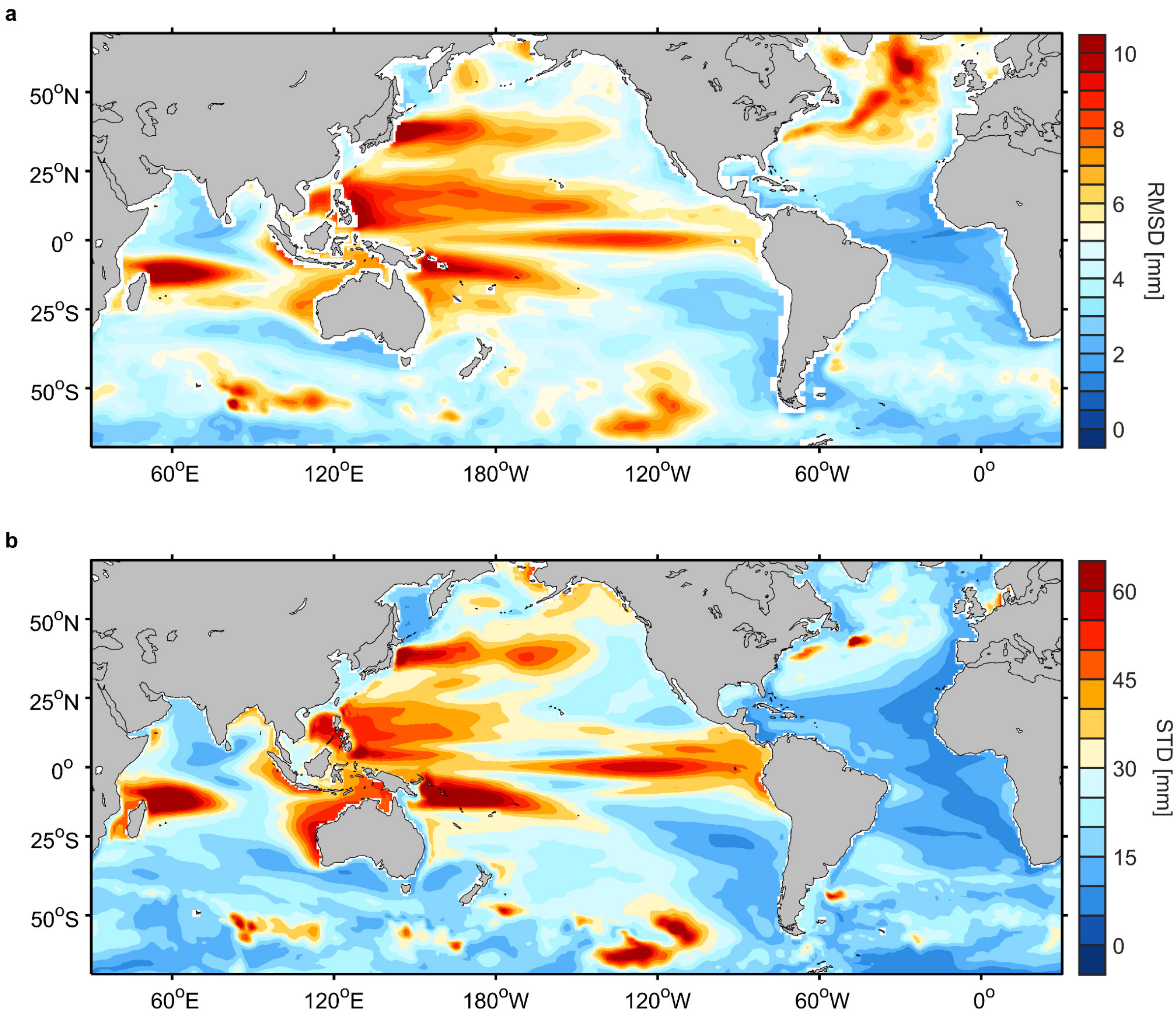

Supplementary Figure 4. Comparing dynamic sea level (DSL) with and without low-pass filtering. a, The root mean square difference (RMSD) [mm] between DSL with and without a 20-year low-pass filtering before deriving multiple-model ensemble mean from the Coupled Model Intercomparison Project Phase 5 (CMIP5) projections under Representative Concentration Pathway (RCP) 4.5 (2007-2100). **b**, standard deviation (STD) [mm] of the de-trended DSL from one typical CMIP5 model CCSM4 over the historical period (1950-2000) under RCP 4.5. Note different colour bars are used for (**a**) and (**b**).

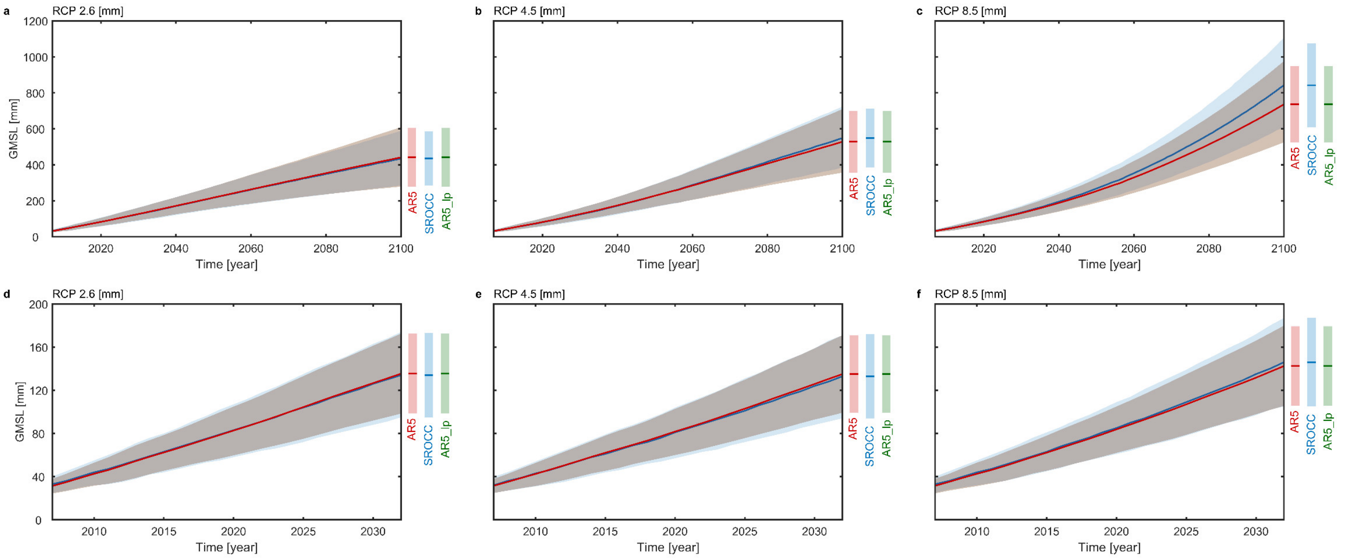

Supplementary Figure 5. The global mean sea level (GMSL) time series from sea-level projections. The annual multi-model averaged GMSL projections are from the Intergovernmental Panel on Climate Change (IPCC) Fifth Assessment Report (AR5), Special Report on the Ocean and Cryosphere in a Changing Climate (SROCC), and the low-pass filtered AR5 projections (AR5_lp). The lines show the median projections [mm] under Representative Concentration Pathway (RCP) 2.6, 4.5 and 8.5 based on AR5 (red), SROCC (blue), and AR5_lp (green) projections over 2007-2100 (**a-c**) and 2007-2032 (**d-f**) respectively. The *likely* range is shown as a shaded band. Vertical bars [mm] at the right sides of each panel represent the ensemble mean and ensemble spread (5 to 95%) of the *likely* (medium confidence) GMSL at the year 2100 (**a-c**) and 2032 (**d-f**) respectively.

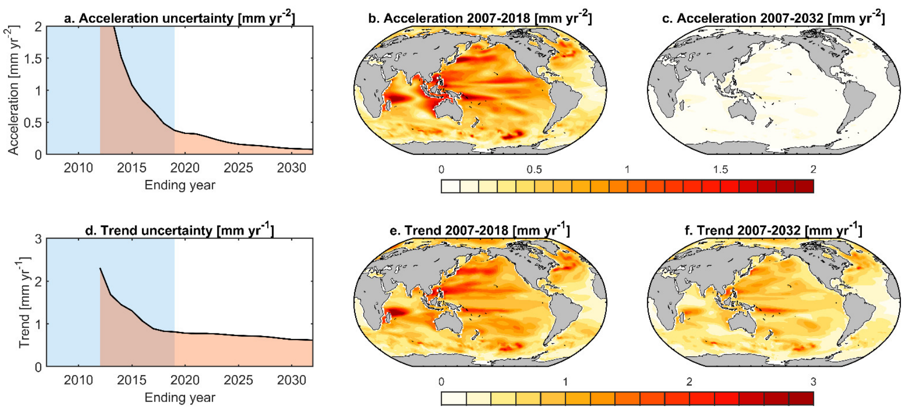

Supplementary Figure 6. Trend and acceleration uncertainties (90% confidence level) of sea level projection. The dynamic sea level (DSL) projections are from the Community Earth System Model (CESM) large ensemble developed by the National Center for Atmospheric Research (NCAR) under Representative Concentration Pathway (RCP) 8.5. The natural variability is removed via multi-model ensemble mean. **a**, The projected acceleration uncertainty [mm yr^{-2}] is estimated by the standard deviation (STD) of 35 subsets of ensemble mean with fixed staring year of 2007 but changing ending years. Values along x-axis indicate the ending year, ranging from 2012 to 2032. The blue shaded area indicates the overlapping period between observations and projections (2007-2018). **b-c**, The spatial map of acceleration uncertainties [mm yr^{-2}] during 2007-2018 and 2007-2032, respectively. **d-f**, Same as (**a-c**) but for linear trend [mm yr^{-1}] during the overlapping period 2007-2032.

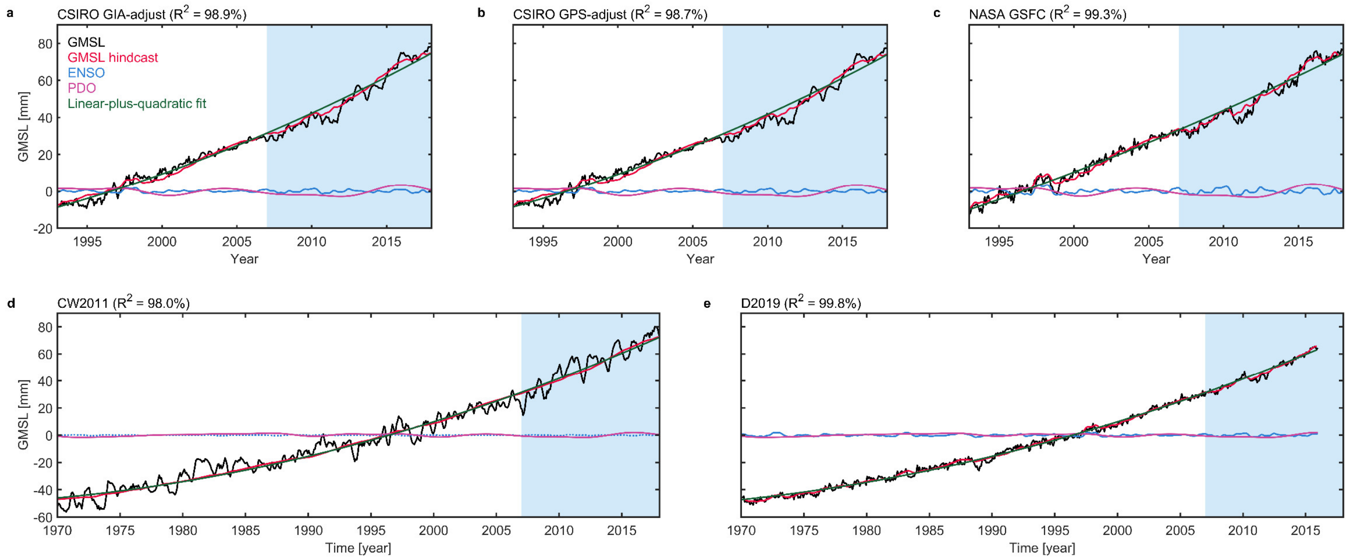

Supplementary Figure 7. The global mean sea level (GMSL) hindcast derived from MVLR model. a-c, Altimeter GMSL observations [mm] from different groups (black), hindcast by multiple variable linear regression (MVLR) model (red, Methods Eq. 1) during 1993-2018. Sea level associated with high-pass filtered El Niño - Southern Oscillation (ENSO; blue) and low-pass filtered PDO (Pacific Decadal Oscillation; purple) climate indices, linear-plus-quadratic fit from the MVLR model (green) are also plotted. **d-e**, Same as (**a-c**), but for GMSL reconstruction [mm] results[1,4]. Dashed line indicates regression coefficient which is not significant at 90% confidence level. The blue shaded area indicates the overlapping period between observations and projections (2007-2018).

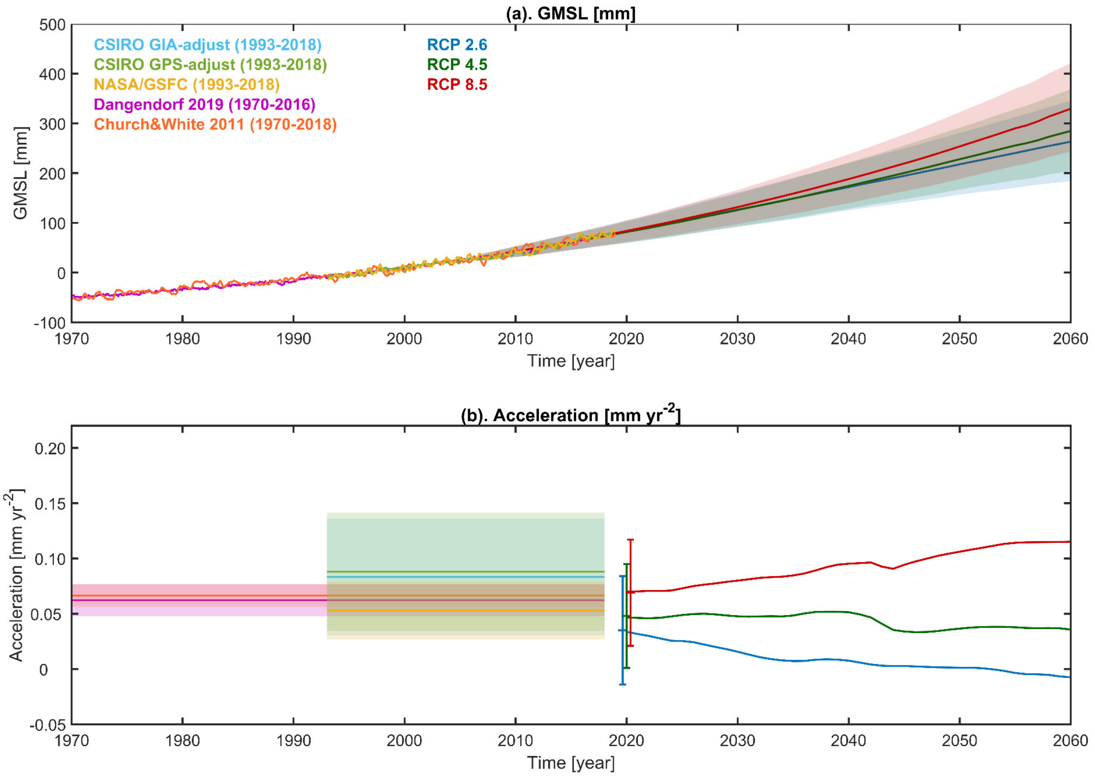

Supplementary Figure 8. Comparing moving acceleration from sea-level projection with observations. a, Time series of global mean sea level (GMSL) [mm] from observations and the Intergovernmental Panel on Climate Change (IPCC) Fifth Assessment Report 5 (AR5) projections under Representative Concentration Pathway (RCP) 2.6, RCP 4.5 and RCP 8.5 scenarios. The shaded region is the likely range of projection. **b**, Boxes denote accelerations [mm yr^{-2}] derived over 1993-2018 from satellite observations and over 1970-2018 from GMSL reconstructions with 90% confidence level, with natural variability removed using multiple variable linear regression (MVLR) model. Lines starting from 2020 indicate projected acceleration derived from 25-year moving quadratic fits under three RCP scenarios. Error bars denote the projected acceleration uncertainties during our research period (2007-2032) with 90% confidence level.

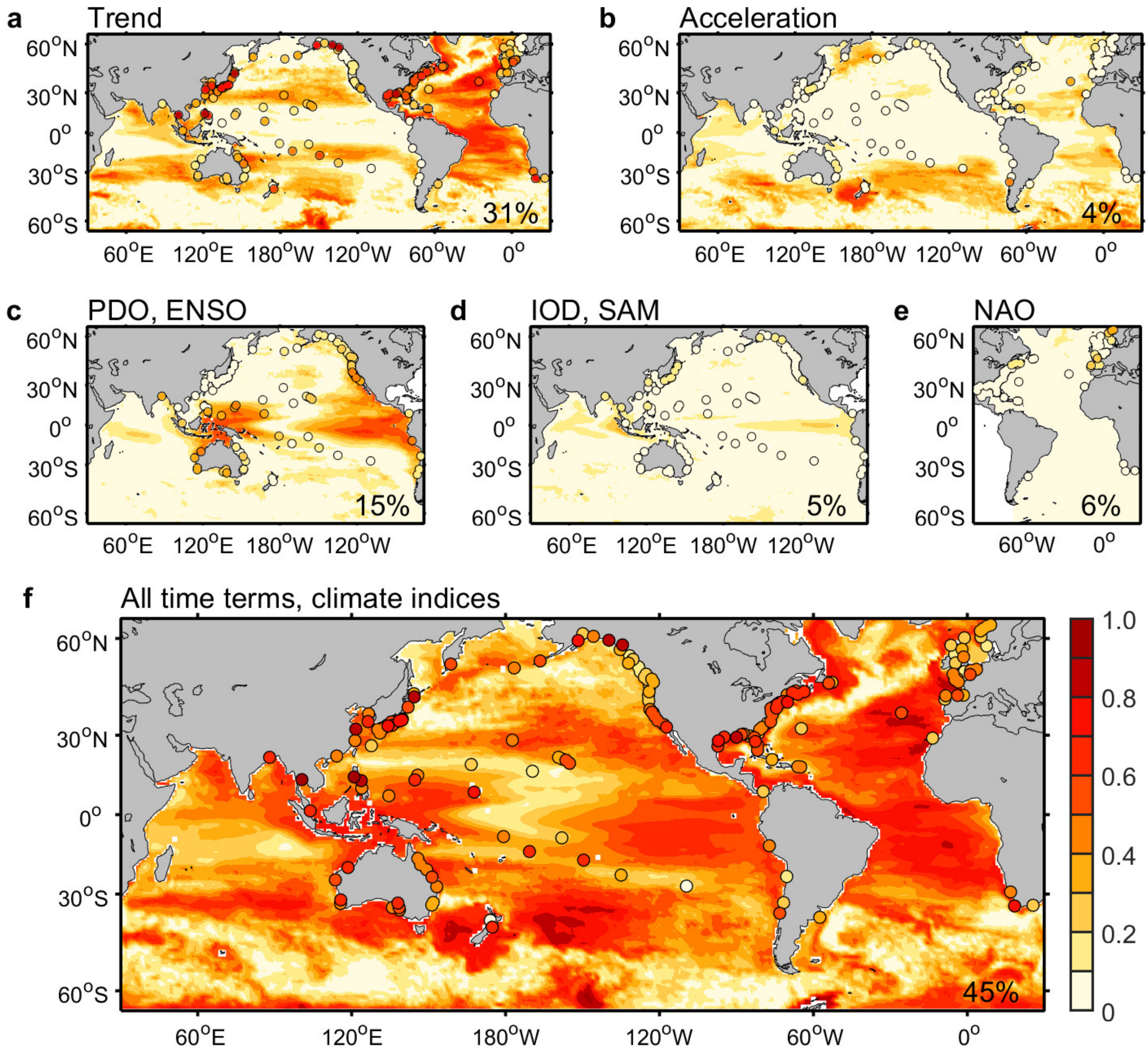

Supplementary Figure 9. Ratio of variance explained by regression over total variance of sea level (R^2). Colored circles indicate R^2 [1] at each tide gauges related to **a,** linear trend, **b**, acceleration, **c**, Pacific Decadal Oscillation (PDO) and El Niño - Southern Oscillation (ENSO), **d**, Indian Ocean Dipole (IOD) and Southern Annular Mode (SAM), **e**, North Atlantic Oscillation (NAO), and **f**, all time terms and climate indices over 1970-2018. The underlying explained variance contours [1] are based on the European Centre for Medium-Range Weather Forecast (ECMWF) Ocean Reanalysis System 5 (ORAS5) reanalysis over the same period. In each panel, the number shown in the lower right corner is the mean explained variance [%] of all tide-gauge stations located in the corresponding region.

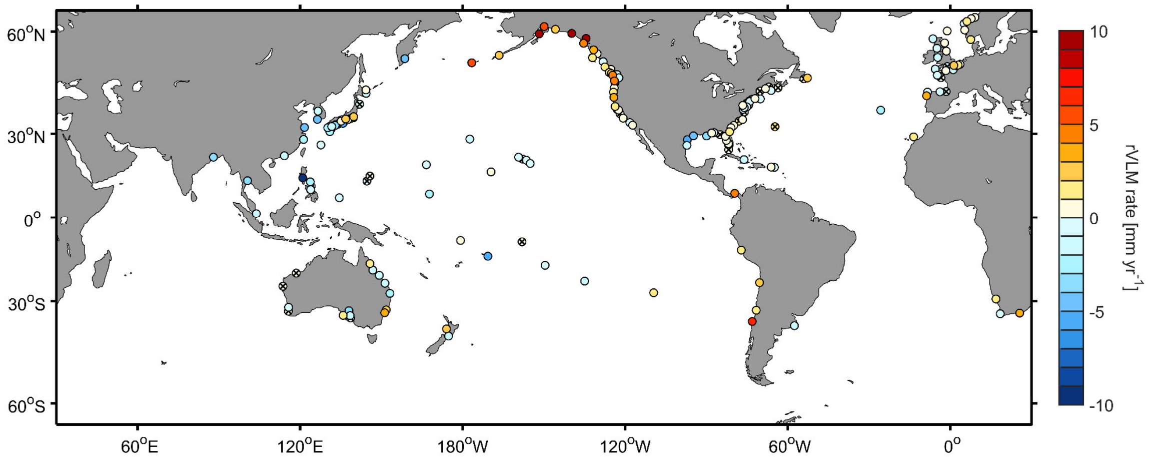

Supplementary Figure 10. Spatial distribution of residual vertical land motion (rVLM) rates [mm yr^{-1}] over 1993-2018. Negative (positive) values denote subsidence (uplift). Trends which are not significant at 90% confidence level are denoted as cross symbols.

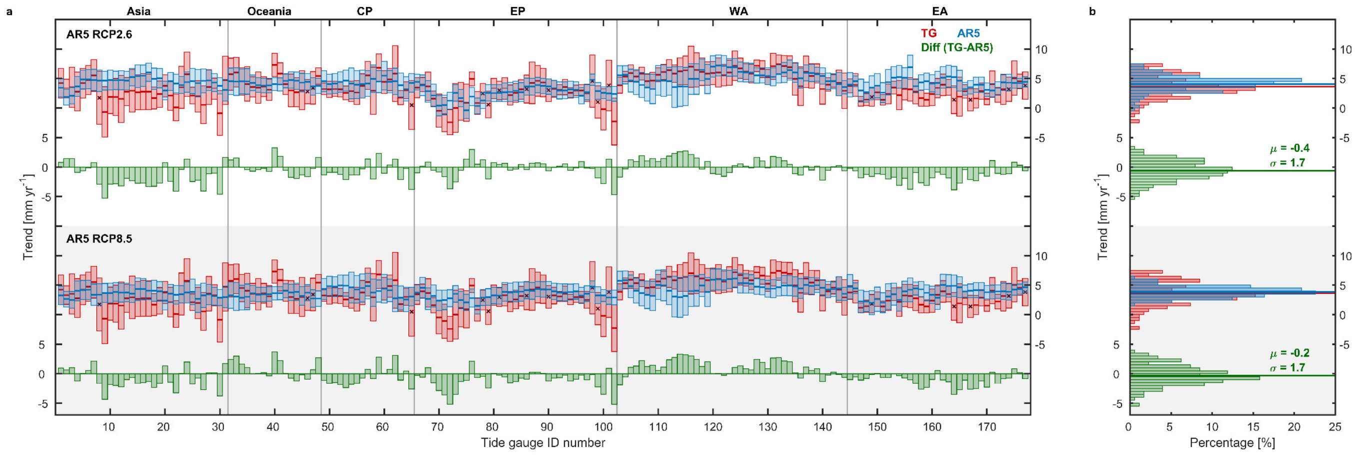

Supplementary Figure 11. Regional sea-level trends over 2007-2018 [mm yr^{-1}] from tide-gauge observations compared with sea-level projections. Sea-level trends [mm yr^{-1}] based on tide gauge observations (TG; red), the Intergovernmental Panel on Climate Change (IPCC) Fifth Assessment Report (AR5) projection (blue) and their difference (TG minus AR5; green) at each tide gauge station, for (**a**) box plots and (**b**) histogram, with AR5 under Representative Concentration Pathway (RCP) 2.6 (top panel) and 8.5 (bottom panel). Error bars indicate 90% confidence level, and trends which are not significant at 90% confidence level denoted as cross symbols. Region definition in **a** is shown in Fig. 2. TG trends have residual vertical land motion (rVLM) adjustment and climate variability removed via multiple variable linear regression (MVLR) model in bottom panel. In histograms **b**, the bin width is 0.5 mm yr^{-1}, horizontal lines present weighted mean trend (μ) at all TG stations, σ denotes the standard deviation of the trend at all TGs.

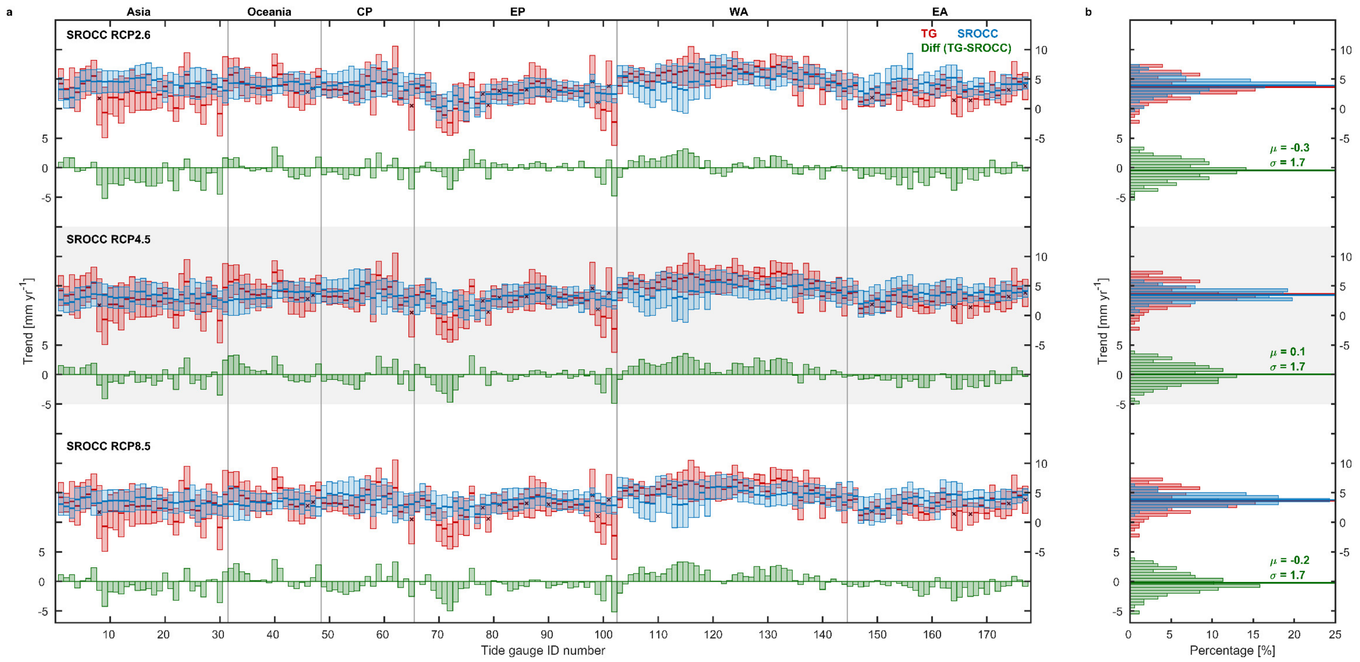

Supplementary Figure 12. Regional sea-level trends over 2007-2018 [mm yr^{-1}] from tide-gauge observations compared with sea-level projections. Sea-level trends [mm yr^{-1}] based on tide gauge observations (TG; red), the Intergovernmental Panel on Climate Change (IPCC) Special Report on the Ocean and Cryosphere in a Changing Climate (SROCC) projection (blue) and their difference (TG minus SROCC; green) at each tide gauge station, for (**a**) box plots and (**b**) histogram, with AR5 under Representative Concentration Pathway (RCP) 2.6 (top panel), 4.5 (middle panel) and 8.5 (bottom panel). Error bars indicate 90% confidence level, and trends which are not significant at 90% confidence level denoted as cross symbols. Region definition in **a** is shown in Fig. 2. TG trends have residual vertical land motion (rVLM) adjustment and climate variability removed via multiple variable linear regression (MVLR) model in bottom panel. In histograms **b**, the bin width is 0.5 mm yr^{-1}, horizontal lines present weighted mean trend (μ) at all TG stations, σ denotes the standard deviation of the trend at all TGs.

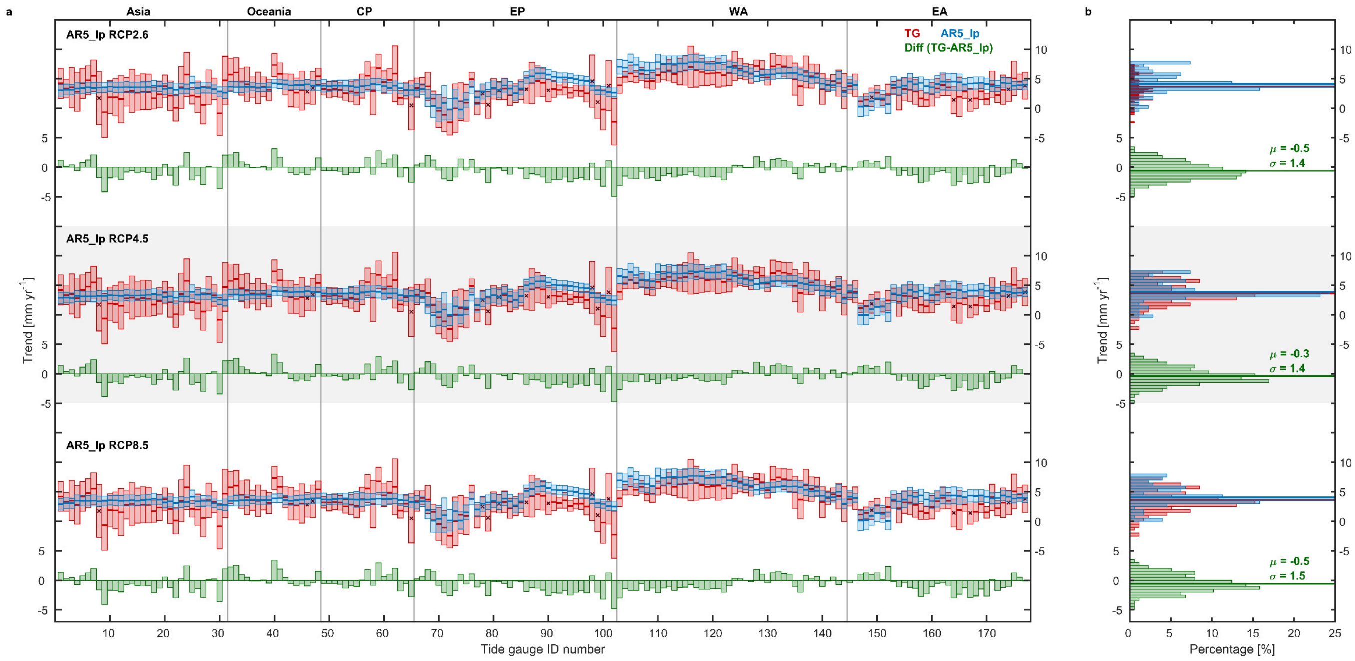

Supplementary Figure 13. Regional sea-level trends over 2007-2018 [mm yr^{-1}] from tide-gauge observations compared with sea-level projections. Same as **Supplementary Fig. 12**, but comparing with the Intergovernmental Panel on Climate Change (IPCC) Fifth Assessment Report projections with 20-year running-mean low-pass filtering (AR5_lp).

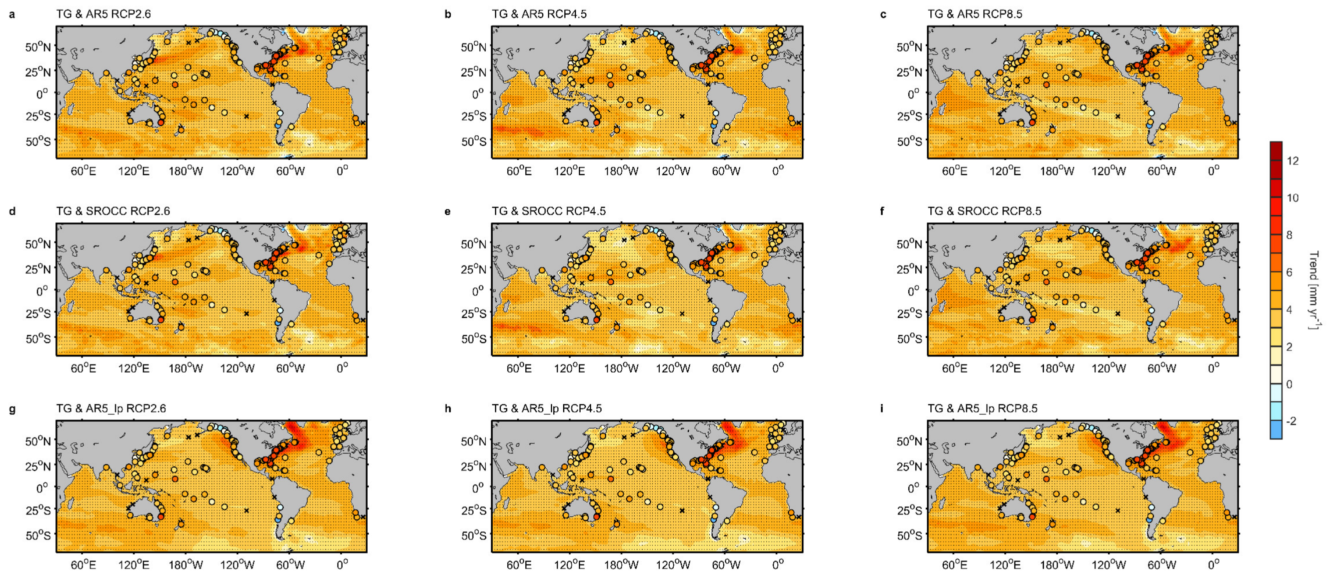

Supplementary Figure 14. Spatial trend patterns from tide-gauge observations compared with sea-level projections over the common period (2007-2018). Coloured circles indicate tide gauge (TG) trends [mm yr^{-1}] with the residual vertical land motion (VLM) adjustment and climate variability removed via multiple variable linear regression (MVLR) model. The underlying trend maps [mm yr^{-1}] are based on the Intergovernmental Panel on Climate Change (IPCC) Fifth Assessment Report (AR5; **a-c**), Special Report on the Ocean and Cryosphere in a Changing Climate (SROCC; **d-f**) and the low-pass filtered AR5 projections (AR5_lp; **g-i**) under three Representative Concentration Pathway (RCP) 2.6, 4.5 and 8.5 scenarios respectively. Tide-gauge trends which are not significant at 90% confidence level denote as cross symbols. Stippling indicates where the projected trends are statistically significant at the 90% confidence level.

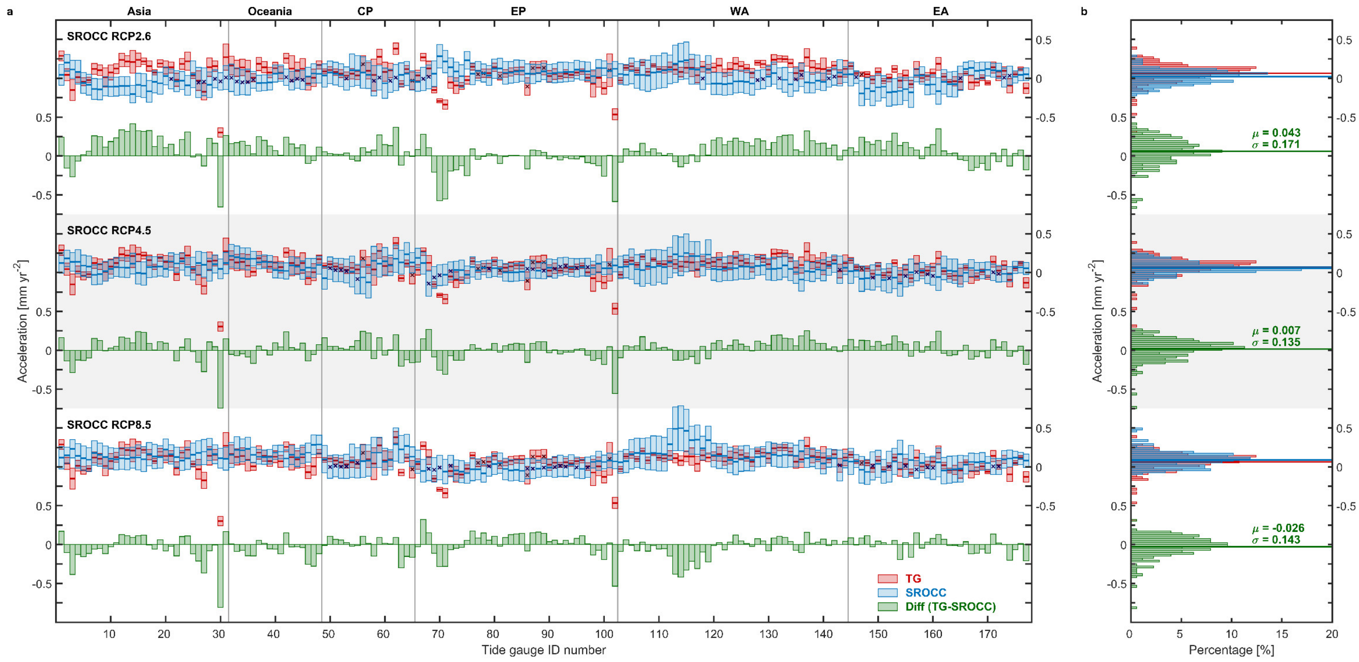

Supplementary Figure 15. Regional sea-level accelerations [mm yr^{-2}] from tide-gauge observations (1970-2018) compared with sea-level projections (2007-2032). Same as **Fig. 6**, but comparing with the Intergovernmental Panel on Climate Change (IPCC) Special Report on the Ocean and Cryosphere in a Changing Climate (SROCC) projections.

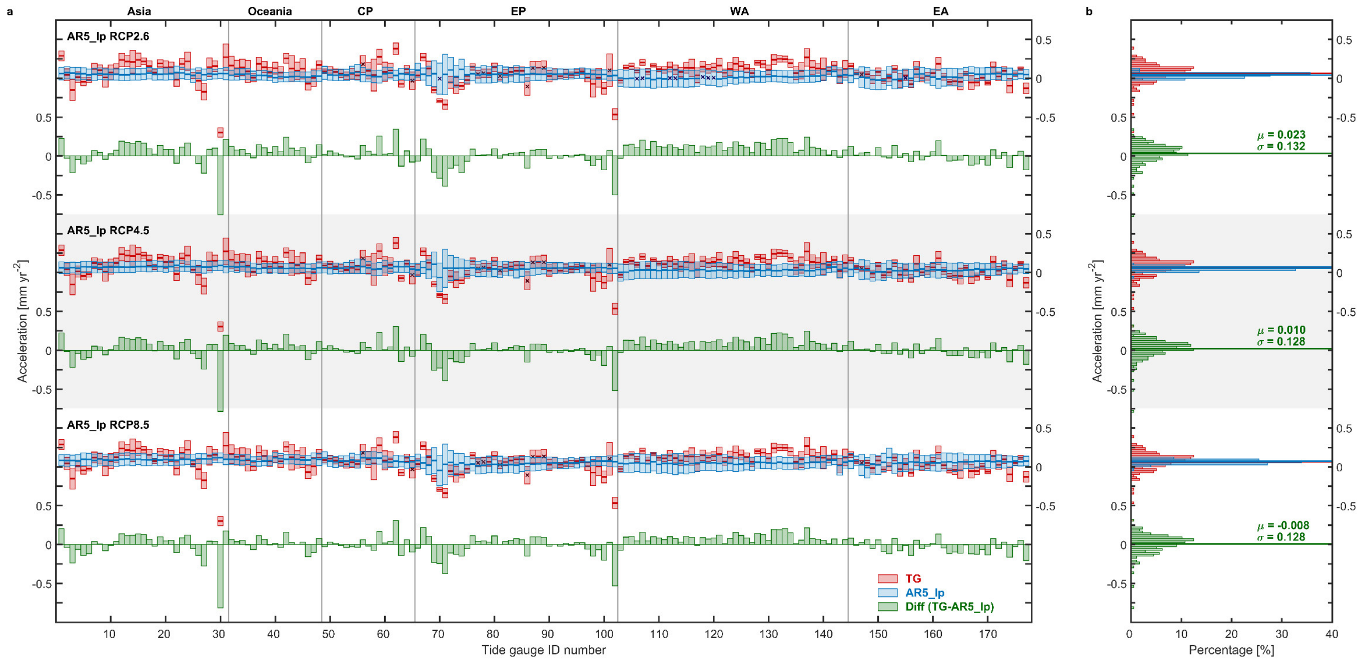

Supplementary Figure 16. Regional sea-level accelerations [mm yr^{-2}] from tide-gauge observations (1970-2018) compared with sea-level projections (2007-2032). Same as **Fig. 6**, but comparing with the Intergovernmental Panel on Climate Change (IPCC) Fifth Assessment Report projections with low-pass filtering (AR5_lp).

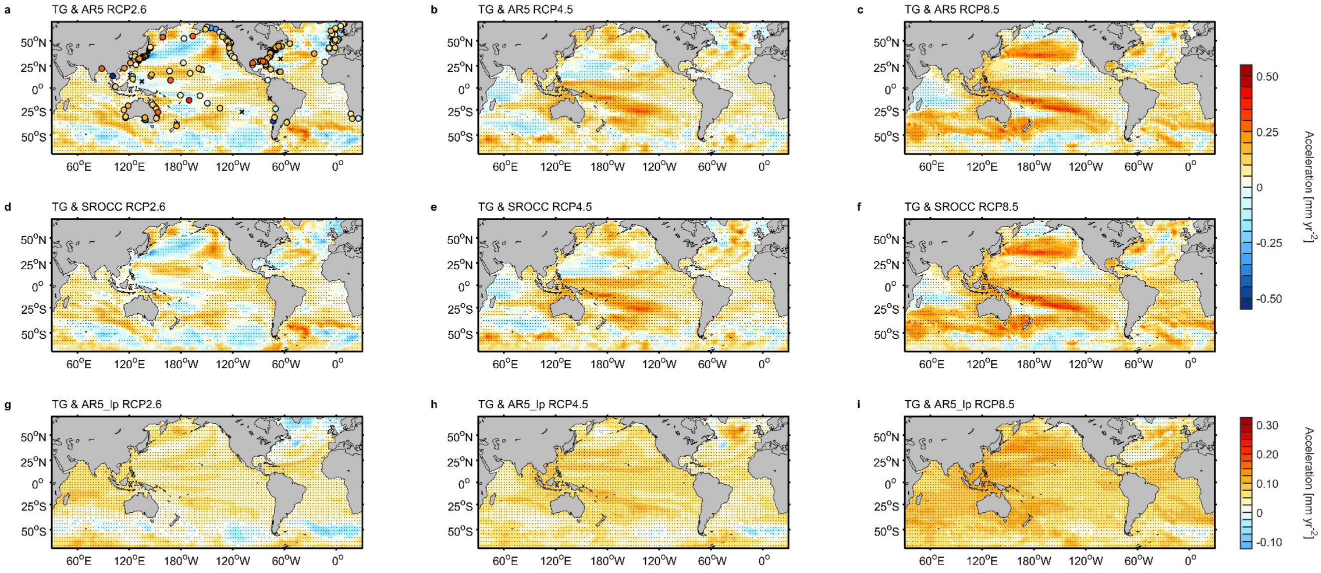

Supplementary Figure 17. Spatial acceleration [mm yr^{-2}] patterns from tide-gauge observations (1970-2018) compared with sea-level projections (2007-2032). Same as **Supplementary Fig. 14**, but for accelerations. Note there is a scale difference between (**a-f**) and (**g-i**).

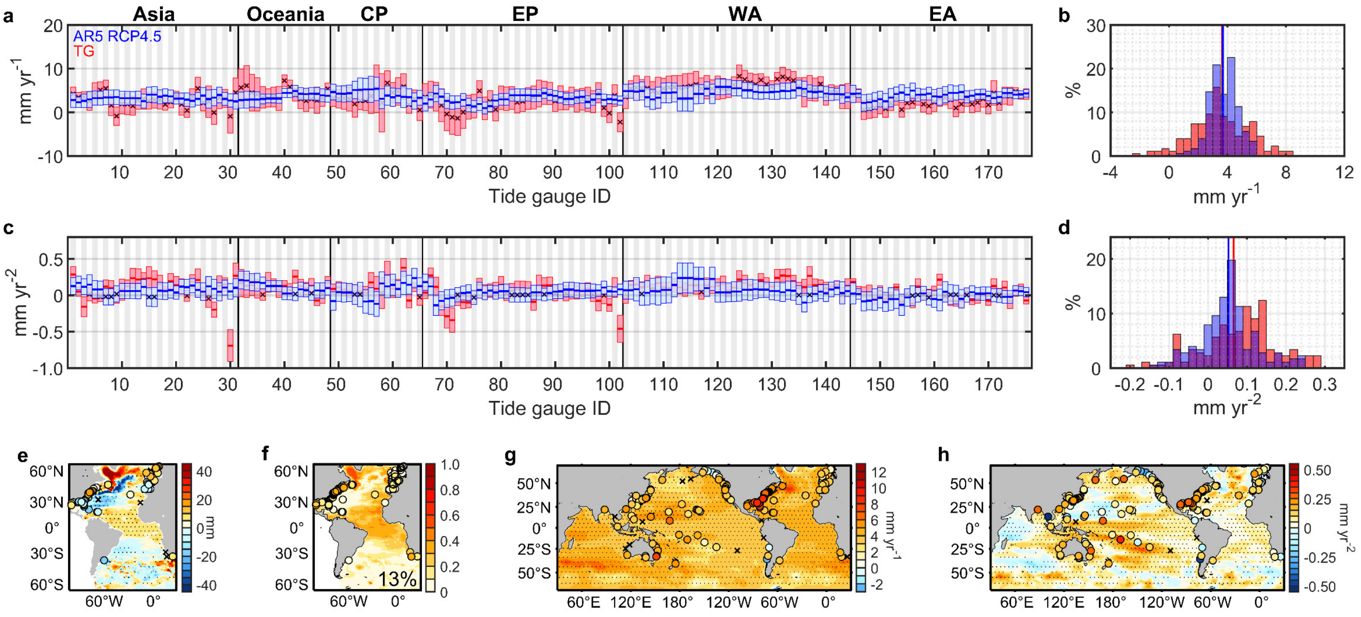

Supplementary Figure 18. Results when the Atlantic Meridional Overturning Circulation (AMOC) index is included in the multiple variable linear regression (MVLR) analysis. a-b, Regional sea-level trends [mm yr^{-1}] over 2007-2018 from tide-gauge observations (TG) compared with the Intergovernmental Panel on Climate Change (IPCC) Fifth Assessment Report (AR5) projection under Representative Concentration Pathway (RCP) 4.5. **c-d,** Regional sea-level acceleration [mm yr^{-2}] from tide-gauge observations (1970-2018) compared with AR5 projection under RCP 4.5 (2007-2032). Region definition is shown in Fig. 2. **e**, Regression coefficient [mm] pattern related to AMOC. **f**, R^2 [1] map related to AMOC. **g**, Spatial trend [mm yr^{-1}] patterns over 2007-2018 from tide-gauge observations compared with sea-level projections under RCP 4.5. **h**, Spatial acceleration [mm yr^{-2}] patterns from tide-gauge observations (1970-2018) compared with sea-level projections under RCP 4.5 (2007-2032).

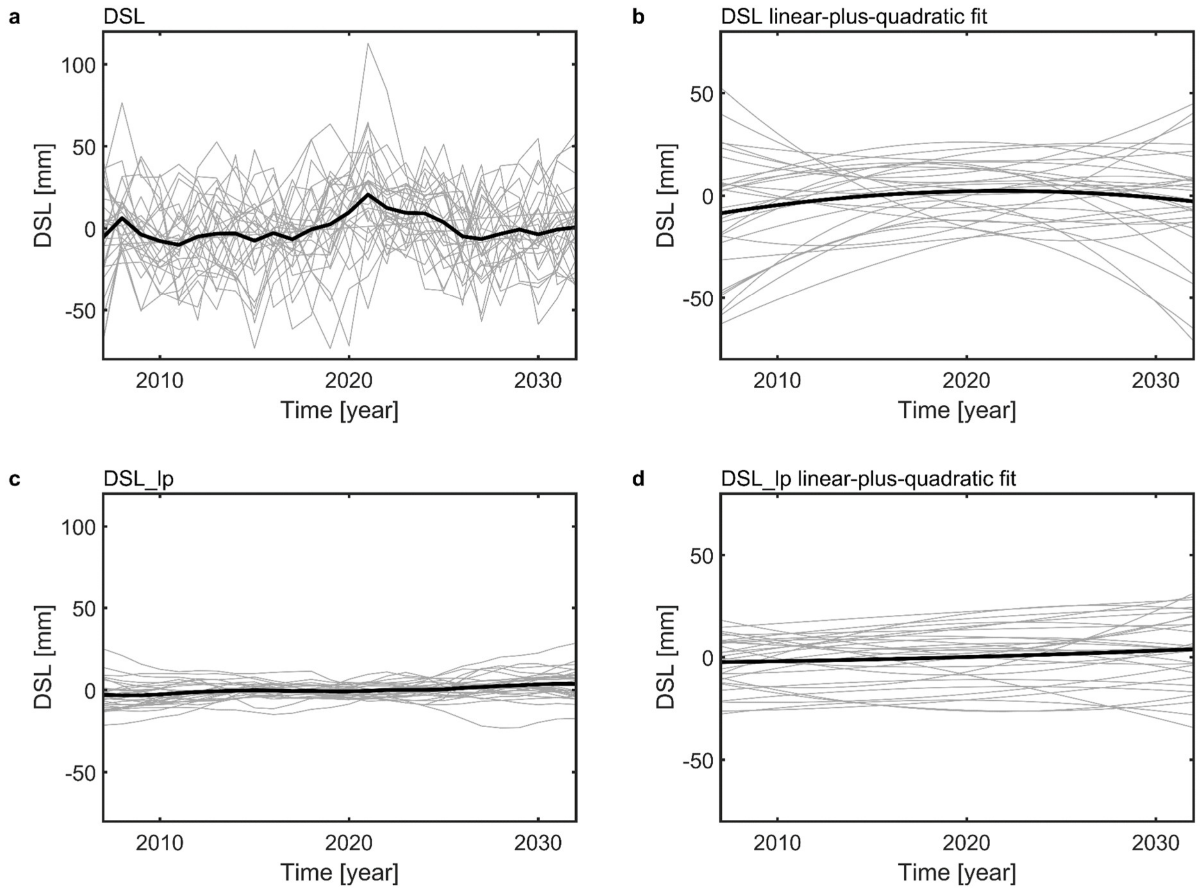

Supplementary Figure 19. Comparing dynamic sea level (DSL) with and without low-pass filtered DSL near one example tide-gauge station. a, Annual time series [mm] and **b**, Linear-plus-quadratic fit [mm] of DSL near TG identification number 90 (location in **Fig. 2**). The grey lines present outputs from 28 Coupled Model Intercomparison Project Phase 5 (CMIP5) models under Representative Concentration Pathway (RCP) 4.5, with the black line showing the ensemble mean. **c-d**, Same as (**a-b**), except for each individual model applied 20-year running mean before further analysis.

Supplementary References

1. Church, J. A. & White, N. J. Sea-level rise from the late 19th to the early 21st century. *Surv. Geophys.* **32**, 585-602 (2011).
2. Jevrejeva, S., Moore, J. C., Grinsted, A., Matthews, A. P. & Spada, G. Trends and acceleration in global and regional sea levels since 1807. *Glob. Planet. Change* **113**, 11-22 (2014).
3. Hay, C. C., Morrow, E., Kopp, R. E. & Mitrovica, J. X. Probabilistic reanalysis of twentieth-century sea-level rise. *Nature* **517**, 481 (2015).
4. Dangendorf, S. et al. Persistent acceleration in global sea-level rise since the 1960s. *Nat. Clim. Change* **9**, 705-710 (2019).
